# Supplementary material for: Conditional chemoconnectomics (cCCTomics) as a strategy for efficient and conditional targeting of chemical transmission
Source: eLife. 2024 Apr 30;12:RP91927. doi: 10.7554/eLife.91927 (PMC11060718; doi:10.7554/eLife.91927)
Supplement: Figure 3—source data 3. [file elife-91927-fig3-data3.zip › Figure 3 source data3 part 2.zip!/Figure 3-Source data 3-part2/1002-9-CRISPResso_Report_e53dd8m5.zip!/Nucleotide_percentage_quilt_around_sgRNA_GTATCTGTCCAGCTACGTGA.pdf]

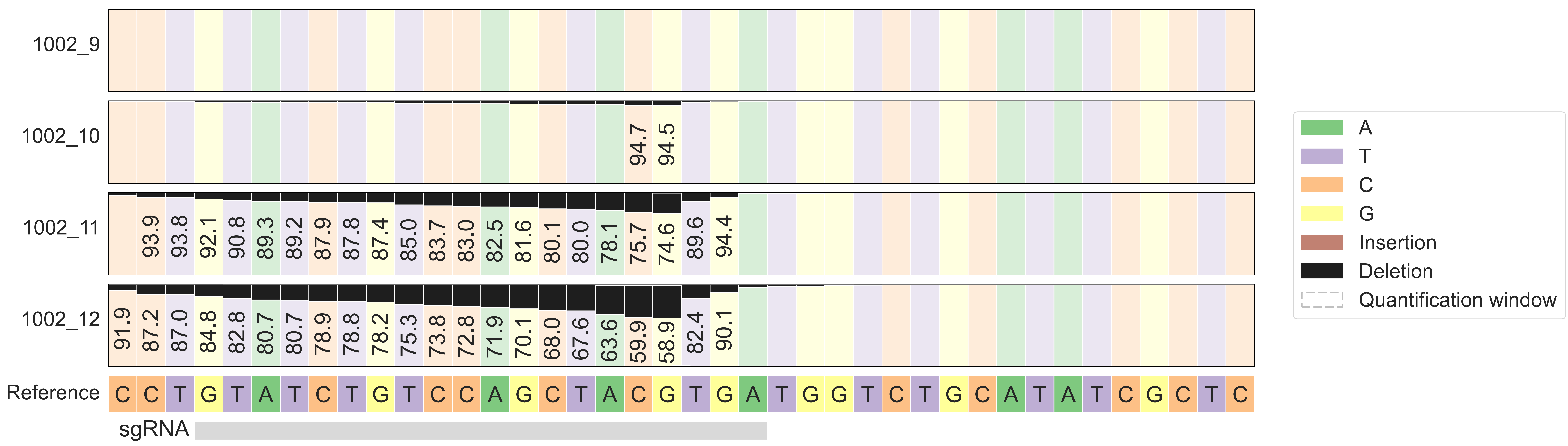
1002_9
1002_10
94.7
94.5
1002_11
93.9 93.8 92.1 90.8 89.3 89.2 87.9 87.8 87.4 85.0 83.7 83.0 82.5 81.6 80.1 80.0 78.1 75.7 74.6 89.6 94.4
1002_12
91.9 87.2 87.0 84.8 82.8 80.7 80.7 78.9 78.8 78.2 75.3 73.8 72.8 71.9 70.1 68.0 67.6 63.6 59.9 58.9 82.4 90.1
Reference
C C T G T A T C T G T C C A G C T A C G T G A T G G T C T G C A T A T C G C T C
sgRNA
A
T
C
G
Insertion
Deletion
Quantification window
